# Precision psychiatry in psychosis - clinicians’ perspective

**DOI:** 10.1192/j.eurpsy.2026.10403

**Published:** 2026-07-31

**Authors:** M. Rojnic Kuzman

**Affiliations:** 1 Zagreb school of medicine; 2 Zagreb University Hospital Centre, Zagreb, Croatia

## Abstract

**Abstract:**

In clinical practice and research, the terms *psychosis* and *schizophrenia* are used to describe clinical presentations characterized by a constellation of positive, negative, and cognitive symptoms persisting for longer than one month. Despite shared diagnostic criteria, these disorders are highly heterogeneous with regard to etiology, phase-specific clinical manifestations, illness trajectory, treatment response, and overall outcome. This heterogeneity presents substantial challenges for both clinical management and research.

Over the past two decades, extensive research has focused on identifying biomarkers across multiple domains, including genomics, epigenetics, neuroimaging, transcriptomics, and, more recently, artificial intelligence. The overarching goal of these efforts has been to enable risk prediction and early detection, enhance diagnostic accuracy, facilitate individualized treatment strategies, improve monitoring, and predict treatment outcomes.

These advances have contributed to the emergence of precision psychiatry, which—similarly to other medical specialties—aims to support clinical decision-making and ultimately improve outcomes for individuals with psychosis and schizophrenia. However, most of these innovations remain confined to research settings and have yet to be systematically implemented or validated in routine clinical practice.

In this presentation, we will review the existing tools that show the greatest promise for successful clinical implementation from the perspective of practicing psychiatrists, in alignment with the EPA viewpoint on Precision Medicine in Mental Health: Applications, Challenges, and Recommendations developed by the EPA Task Force Precision Psychiatry.

**Disclosure of Interest:**

None Declared

